# Caregiver Mastery as a Mediator Between Dementia Behavioral and Psychological Symptoms and Depressive Symptoms

**DOI:** 10.1093/geroni/igaf122.3725

**Published:** 2025-12-31

**Authors:** Kyungmi Lee, Faith Jackson, Kylie Meyer

**Affiliations:** Case Western Reserve University, Cleveland, Ohio, United States; Case Western Reserve University, Cleveland, Ohio, United States; Case Western Reserve University, Cleveland, Ohio, United States

## Abstract

Self-efficacy is an individual’s belief in their ability to handle challenging situations. Caregivers who experience greater bother from behavioral and psychological symptoms (BPSD) of dementia are at high risk of experiencing depressive symptoms. While many studies have explored the impact of BPSD on caregivers’ depression, limited research has examined how caregivers’ self-efficacy may buffer the negative effects of BPSD. This study investigates whether caregiver self-efficacy, and the related concepts mastery and confidence (i.e., care tasks, decision-making, and knowledge), mediate the relationship between BPSD and caregivers’ depression. Mediation analysis was performed using baseline data from an ongoing clinical trial with 130 caregivers, and indirect effects were assessed via bootstrapping with 95% confidence intervals. Results show that mastery significantly mediates the effect of BPSD on depressive symptoms, even after controlling for caregiver age, sex, and caregiving duration. Higher BPSD was associated with lower mastery (β = -0.066, SE = 0.019, p = .0009); lower mastery was also associated with higher depressive symptoms (β = -0.400, SE = 0.118, p = .0010). The indirect effect of mastery was statistically significant (β = 0.027, SE = 0.011, 95% CI [0.009, 0.051]). Among covariates, sex was positively associated with mastery (β = 0.216, p = .013), while age and caregiving duration were not significantly related to mastery or depressive symptoms. Other self-efficacy components did not show significant mediation effects. These findings support mastery as an independent mediator between BPSD and caregiver depressive symptoms, highlighting the importance of interventions targeting mastery to reduce depression in caregivers.

